# Structural and functional connectivity of vestibular graviceptive to sensory and motor circuits

**DOI:** 10.1093/braincomms/fcaf290

**Published:** 2025-08-08

**Authors:** Julian Conrad, Laurenz Eberle, Bernhard Baier, Rainer Boegle, Marianne Dieterich, Andreas Zwergal

**Affiliations:** Department of Neurology and German Center for Vertigo and Balance Disorders, LMU University Hospital, LMU Munich, Munich 81377, Germany; Division for Neurodegenerative Diseases, Department of Neurology, Universitaetsmedizin Mannheim, Heidelberg University, Theodor-Kutzer-Ufer 1-3, Mannheim 68167, Germany; Department of Neurology and German Center for Vertigo and Balance Disorders, LMU University Hospital, LMU Munich, Munich 81377, Germany; Department of Radiology, Augsburg University Hospital, University of Augsburg, Augsburg 86156, Germany; Department of Neurology, Edith Stein Fachklinik Bad Bergzabern, Bad Bergzabern 76887, Germany; Department of Neurology and German Center for Vertigo and Balance Disorders, LMU University Hospital, LMU Munich, Munich 81377, Germany; Department of Neurology and German Center for Vertigo and Balance Disorders, LMU University Hospital, LMU Munich, Munich 81377, Germany; Graduate School of Systemic Neurosciences (GSN), LMU Munich, Munich 81377, Germany; SyNergy—Munich Cluster for Systems Neurology, Munich 81377, Germany; Department of Neurology and German Center for Vertigo and Balance Disorders, LMU University Hospital, LMU Munich, Munich 81377, Germany; Graduate School of Systemic Neurosciences (GSN), LMU Munich, Munich 81377, Germany

**Keywords:** balance, lesion-network mapping, subjective visual vertical (SVV), basal ganglia, cortical

## Abstract

Processing of vestibular graviceptive signals from the inner ear is essential for spatial perception, bipedal stance, locomotion, and navigation in a three-dimensional world. Acute unilateral ischaemic lesions along the central vestibular pathways lead to deficits of gravitational processing which can be quantified as perceptual tilts of the subjective visual vertical (SVV). For ipsiversive and contraversive directional tilts, dichotomous networks were documented from the brainstem to the thalamus. In the current lesion-network mapping study, we asked whether this dichotomy of directional tilts of gravitational processing is maintained at the cortical level. 107 patients with acute right-hemispheric infarcts (mean age 66 years, ±13 years) in the territory of the middle cerebral artery were included in the study. To examine the association of tilts of the SVV with lesion locations, support-vector regression lesion-symptom mapping (SVR-LSM) was used with tilt of the SVV as a continuous variable. Analyses were carried out for ipsi- and contraversive tilts separately. In addition, we performed disconnectome mapping and SVR-LSM-disconnectome analyses by referencing lesions to a normative connectome detect structural networks associated with SVV tilts. Similarly, functional connectivity mapping was used to determine the functional networks associated with SVV tilts. The SVR-LSM with the functional maps revealed the statistical association between SVV tilt and functional networks. The SVR-LSM analysis demonstrated distinct clusters associated with either ipsi- or contraversive SVV tilts. Ipsiversive tilt clusters were centered around the parieto-(retro)-insular opercular cortex [PIVC, retroinsular area (Ri), posterior insular long gyrus (Ig), parietal operculum (OP2-3)]. The contraversive tilt cluster showed additional involvement of the motor system (basal ganglia) and the ventral prefrontal cortex (Brodman area BA44, inferior frontal gyrus). In lesions with ipsiversive tilts, a disconnection of fronto-insular tracts and the arcuate fascicle was found. Contraversive tilt related disconnection was observed in the superior longitudinal fascicle (SLFII) and the medial temporal cortex (perirhinal, entorhinal cortex). Cortico-fugal connections could be traced down via the thalamus to the cerebellum and vestibular nuclei. The functional networks associated with ipsiversive and contraversive tilts showed a similar pattern: more restricted in the core vestibular and ocular motor cortical network for ipsiversive tilts, additional involvement of the motor system for contraversive tilts. Thus, the current data demonstrate partly separated cortical networks for gravitational processing associated with directional SVV tilts. These could imply differential routes of vestibular input for sensory and motor processing.

## Teaser

Conrad *et al*. have studied verticality perception in 107 stroke patients using machine-learning based multivariate lesion-network mapping. They found seperable networks for ipsilateral and contralateral tilts of verticality. These networks could influence sensory control of head/body posture and modulation of the motor system.

## Graphical Abstract

**Graphical Abstract:**
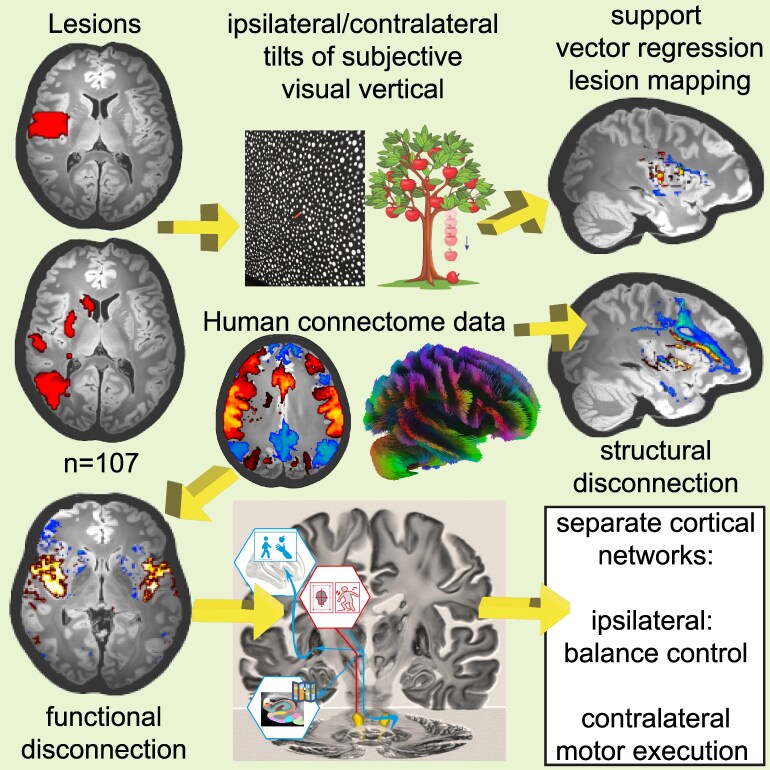

## Introduction

Graviceptive directional input is necessary for the control of bipedal stance, locomotion, and spatial navigation, as well as the computation of self-perception and motor outputs.^1,2^ Human vestibular graviceptors are located in the otolith organs of the inner ear and supported by input from the vertical semicircular canals.^2-5^ The lack of reliable gravitational priors can lead to severe disorders of balance and postural stability.^1^ Apart from the vestibular brainstem pathways for balance control, the thalamo-cortical vestibular network plays a key role in maintaining balance relative to gravity.^6,7^ A disturbance in the thalamo-cortical network has detrimental effects on postural stability. For example, thalamic astasia due to an acute thalamic lesion leads to a strong tendency to fall to one side in the absence of a paresis.^8,9^ Similarly, contraversive pusher syndrome is characterized by a severe transmodal tilt of verticality perception.^10^ It is the consequence of a thalamo-cortical disconnection for multimodal graviceptive processing as was found in thalamic and connected cortical lesions.^11-13^

Furthermore, a dichotomy for vestibular graviceptive processing, with separate pathways and networks for ipsiversive and contraversive graviceptice tilts, has been demonstrated from the brainstem to the thalamus.^14-16^ Recent data showed evidence that these distinct pathways have differential cortical connections.^7^ These findings suggest a functional specialization: (i) vestibular perceptive for the uncrossed pathway to the core vestibular cortical network hubs and (ii) vestibular modulation of motor control for the crossed pathway that connects to the entire somato-motor network.^7^

In the current multimodal imaging study, we tried to answer whether distinct cortical hubs for directional verticality tilts and associated whole-brain networks exist in the right hemisphere (RH).

Machine-learning based multivariate lesion-symptom mapping (support-vector regression lesion-symptom mapping) was used to determine the cortical and subcortical regions that are essential for vestibular graviception. Furthermore, we studied disconnectome mapping to establish the structural and functional (dis-) connectivity pattern of directional cortical graviceptive processing.

Our hypothesis was that separable cortical networks are associated with ipsiversive and contraversive graviceptive vestibular processing.

## Materials and methods

### Patient selection

Prospectively, 107 patients (48 female, 59 male, mean age 66 years) with an acute, first ever cortico-subcortical infarct within the RH MCA-territory were enrolled. Data was collected at Mainz University Hospital and at LMU University Hospital, LMU Munich, Germany. Three patients showed substantial thalamic involvement and were not included in the final analysis (all with contraversive tilts), resulting in a total of 104 patients. Some data from the Mainz cohort have been published previously.^17^

All patients received a dedicated neurological and neuro-otological work-up. This included a detailed neuro-orthoptic assessment including measurements of the subjective visual vertical (SVV) by the hemispheric dome method^14,18^ as an estimate of vestibular graviception.

For SVV measurements, patients sat in complete darkness looking into a hemispheric dome with their head on a chin rest mounted ∼1 m from the visual target. Patients had to adjust a luminous rod projected in the center of the dome to their subjective vertical using a joystick. To minimize visual cues to orientation, a randomized dot pattern was displayed in the half-spherical dome. The rod started with a randomized offset of 10°–40° from the objective vertical to either side with a measurement precision of up to 0.01°. We used the mean of 8 static measurements of SVV obtained under binocular viewing conditions. Here, a mean deviation of > ±2.5° was considered pathological (normal range ± 2 SD ≙ 2.5°).^18^ Negative values indicate left-sided tilts, while values above 0° indicate right-sided tilts. Tilts to the side of the lesion are defined as ipsiversive tilts, tilts to the contralateral side as contraversive tilts.

Neuropsychological testing was performed by means of the paper-pencil neglect tests (bells test^19^), the scale for contraversive pushing,^20,21^ and the Edinburgh handedness inventory. Exclusion criteria were prior ischaemic infarct, severe white matter lesion load (Fazekas >2), reduced level of consciousness, or aphasia with inability to comprehend the study instructions.

### Imaging

All patients received a cranial MRI in the acute phase that included diffusion-weighted imaging [DWI; 3 mm, echo time (TE) = 880 milliseconds, repetition time (TR) = 1400 milliseconds, 48 slices] sequences. Lesions were delineated on the acute phase DWI in MRICROGL (https://www.nitrc.org/projects/mricrogl) and normalized to MNI152 space using the Clinical Toolbox using SPM12 (https://www.fil.ion.ucl.ac.uk/spm/software/spm12/) in Matlab (version 2022a, https://de.mathworks.com).

### Multivariate lesion-symptom mapping

Multivariate support-vector regression lesion-symptom mapping (SVR-LSM) was used to determine the statistical association between tilts of the perception of verticality with specific lesion locations.^22^ SVR-LSM analyses were performed using a non-linear radial basis function kernel as implemented in the SVR-LSM toolbox.^22^ Hyperparameters were kept at the default setting of the SVR-LSM toolbox (cost = 30, *γ* = 5).^23^ For the SVR, lesion maps were vectorized, lesion volume was used as a regressor for the behavioural and lesion data.

Extensive permutation testing (10 000 permutations) was performed to determine a statistical *P*-value threshold on a voxel-level based on the SVR-β values calculated by SVR. False discovery rate (FDR) correction was applied afterwards to the resulting voxelwise-thresholded *P*-value maps (*P* = 0.05) to control for false positives using the FDR function in FSL, FDR was set to 5% (*q* = 0.05) (https://fsl.fmrib.ox.ac.uk/fsl, supplementary table 1 shows the *P*-value-cut-offs of the FDR-correction).^7^

SVV tilts were used as a continuous variable. A conservative lesion threshold of 10% of the sample was applied. The statistical model included hyperparameter optimization, and *k*-fold cross-validation with *k* = 5 iterations was used to optimize model fit. Separate analyses were conducted for lesions that caused ipsiversive and contraversive tilts of the SVV. Cases with an SVV tilt of exactly 0° (*n* = 12) were included in both analyses. All results are depicted with the FDR-corrected significance thresholds on a high-resolution 7T post-mortem MRI template.^24^

### Disconnectome and lesion-network mapping

Historically, lesion-symptom mapping studies were only able to associate direct damage to brain areas with a related symptom. However, functional and structural disconnection of regions distant from the lesion might also be relevant for the occurrence of symptoms. In this regard, both functional and structural disconnection have been used to study distant effects of lesions on brain function.^25,26^ For disconnectome mapping, lesion maps are used as seeds for tractography in a separate dataset of healthy subjects. Streamlines passing through a specific lesion are transformed to visitation maps and the probability of disconnection of white matter tracts is then calculated from these large connectomic datasets. The result is a disconnection probability map that can then be used to explain network effects in patients. Here, it leverages the advantages of average normative connectomes in healthy subjects with high *n* and high-quality tractography data to infer the network dysfunction in the patient group. Therefore, it indirectly assesses voxel-wise white-matter disconnections based on voxelwise disconnection probabilities. This approach has been implemented in the BCB toolkit (http://toolkit.bcblab.com/).^27^

All lesions were used to create disconnectome maps that determine the lesion-specific disconnection probability of white matter tracts using the BCB toolkit. For this, for each lesion-seed a tractography is performed in a separate sample of 180 individuals with high-quality 7T tractography data from the human connectome project (HCP).^26,28^ The patients’ lesions in MNI152 space were registered to the native space of each healthy participant's tractography data using affine and diffeomorphic deformations. This seed was then used to perform the tractography in TrackVis (http://www.trackvis.org/). The results were transformed to visitation maps, binarized, and registered to MNI152 space. From these maps, the percentage of overlap of each voxel in the normalized subject visitation maps was depicted as the voxelwise probability of disconnection for each lesion. The number of voxels within the individual disconnectome map was thresholded at 0.5 (50% or higher probability of disconnection). Tract designation in the BCB-toolkit is based on a dedicated white matter atlas in MNI-space that used state-of-the art spherical deconvolution tractography in comparison to Klingler dissections.^29^

The resulting disconnectome maps were binarized and used as input data for a separate SVR-LSM analysis as described above (i.e. the same control of lesion volume on the imaging and behavioural data was applied).^7,30^ Separate analyses were carried out for disconnection of tracts causing ipsiversive versus contraversive tilts.

The disconnectome maps provide a probability of disconnection in a specific voxel. Allocation of the described cortical areas and of specific white matter tracts in the following maps is based on information from tract atlases and anatomical expertise in cortico-fugal vestibular projections as well as in inter- and intrahemispheric association tracts. However, it is possible that multiple tracts are affected in a specific voxel (see also Supplementary Fig. 5).

The individual normalized lesion maps were used as seeds for the whole-brain resting-state functional connectivity analysis. We used the 100 unrelated subjects preprocessed resting-state functional MRI (fMRI) dataset (54 females, 46 males, mean age = 29.1 ± 3.7 years) from the HCP (https://www.humanconnectome.org/; release Q3). The resting-state fMRI data were acquired as part of the HCP 900 data release using a gradient-echo echo planar imaging sequence (TR = 720 ms, TE = 33.1 ms, flip angle = 52°, field of view = 208 × 180, matrix size = 104 × 90, 2 mm^3^ isotropic, 72 slices, multiband factor = 8, echo spacing = 0.58 ms, band width = 2290 Hz/pixel, 2 sessions, 1200 volumes). Axial oblique acquisitions alternated between phase encoding in a right-to-left and left-to-right direction.^31^ Data were processed following the HCP functional preprocessing guidelines.^32,33^ Processing included artefact removal, motion correction, and registration to standard Montreal Neurological Institute space in volumetric format (MNI152 space).^33^ The functional connectivity analysis was performed using CONN toolbox version 22.a (http://www.nitrc.org/projects/conn) for MATLAB (version 2022a).^34^ For denoising, the following time series were used as covariates in a nuisance regression model; white matter and CSF confounds were each considered with their first 5 principal components. Furthermore, 6 principal temporal components of the movement parameters (3 translation and 3 rotation parameters) were used. Images were denoised with a temporal band-pass filter (0.008–0.09 Hz).

The voxel values in the resulting functional connectivity (fc) maps represent the correlation between the time course of the seed region (i.e. the lesion) and the rest of the brain. SVR-LSM analyses were carried out for correlated networks based on the unthresholded lesion-specific functional connectivity maps controlling for lesion volume as described above.

### Statistical analysis of demographic data

Statistical analyses of clinical and demographic data were performed with SPSS version 29.0.2.0 (https://www.ibm.com/de-de/products/spss-statistics). Group differences were determined between the ipsiversive and contraversive tilts of the SVV. Normal distribution of the data was tested using the Kolmogorov-Smirnov-Test. If the criteria for normal distribution were not met, medians, interquartile ranges (IQR) and minima/maxima were reported. Group differences were determined using the Mann–Whitney U-test with an *α*-threshold of 0.05. For group differences in the distribution of dexterity (handedness), a *X*^2^-test was performed.

### Patient consent and data availability

The study was performed in accordance with the 1964 Declaration of Helsinki, latest adaption Fortaleza 2013. The study was approved by the institutional review boards of LMU University Hospital, LMU Munich, Germany, and the University of Mainz, Germany. All patients gave informed written consent to participate in the study.

## Results

### Clinical characteristics

**Figure 1:**
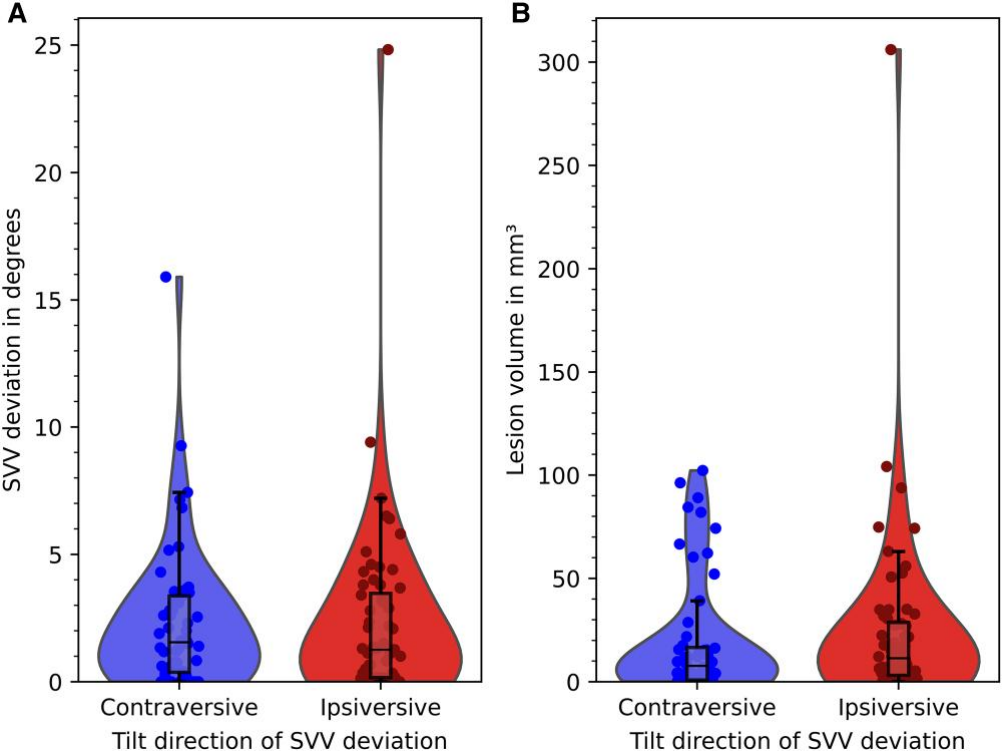
**(A) Violin plot of the distribution of SVV values for contraversive (*n* = 44) and ipsiversive (*n* = 48) tilts, 12 patient had a tilt of 0°. (B) Distribution of lesion volumes among patients with contraversive (blue) and ipsiversive (red) tilts.**  *Y*-axis shows SVV deviation in degrees in A and lesion volume in mm^3^ in B. *X*-axis shows plots for contraversive and ipsiversive SVV tilts. Integrated box plots indicate median and interquartile range (IQR), whiskers show the range of 1.5 × IQR. Dots represent individual measurements. Median SVV tilts and lesion volumes were not significantly different between both groups (Mann–Whitney U-test, *U* = 966; standard error: 130; *z* = 0.862; *P* = 0.716 for median tilt, Mann–Whitney U-test, *U* = 1027; standard error 144, *z* = ; −1.5p = 0.272 for median lesion volume). SVV, subjective visual vertical.

Key demographic data of the patients are presented in Table 1. All patients had right-hemispheric lesions. Patients with SVV measures of exactly 0° (*n* = 12) were considered in the analysis of ipsiversive and contraversive tilts of the SVV, i.e. in both analysis groups. Therefore, the ipsiversive tilt group included 60 patients (48 patients with directional tilt and 12 patients with 0° tilt of the SVV), the contraversive tilt group included 56 patients (44 patients with directional tilt and 12 patients with 0° tilt of the SVV). A total of 43 patients (41.35%) showed pathological tilts of SVV of ≥2.5° to either side (*n* = 21 with ipsiversive tilt, *n* = 22 with contraversive tilt). Median values of absolute SVV tilt were 1.26° (IQR = 3.3°) in ipsiversive tilts and 1.55° (IQR = 3.0°) in contraversive tilts without a significant difference (Mann–Whitney U-test, *P* = 0.716) (Fig. 1). No significant difference was shown in the size of lesion volumes between the two groups (Mann–Whitney U-test, *P* = 0.272).

**Table 1:** Clinical and demographic data

	Ipsiversive tilt (*n* = 60)	Contraversive tilt (*n* = 56)	*P*-value
**Demographic data**			
Age, median years (range, IQR)	68.0 (36.0–89.0, 19.5)	67.0 (33.0–86.0, 19.8)	0.840^a^
Gender (%)			0.489^a^
Female	24 (40.0)	26 (46.4)	
Male	36 (60.0)	30 (53.6)	
**Clinical data**			
Lesion volume, median in ml (range, IQR)	11.4 (0.1–306.0, 25.5)	7.7 (0.1–102.2, 15.7)	0.272^a^
SVV, median degrees (range, IQR)	1.3 (0.0–24.8, 3.3)	1.6 (0.0–15.9, 3.0)	0.716^a^
Handedness (%)			0.463^b^
Right	52 (86.7)	50 (89.3)	
Left	7 (11.7)	5 (8.9)	
Ambidextrous	1 (1.7)	0 (0.0)	
**Symptoms**			
Contralateral paresis (%)	45 (75.0)	41 (73.2)	0.680^b^
Contralateral somatosensory deficit (%)	56 (93.3)	50 (89.3)	0.503^b^
Visual Neglect (%)	12 (20.0)	19 (33.9)	0.092^b^
Pusher-Syndrome (%)	10 (16.7)	13 (23.2)	0.381^b^

Clinical and demographic data of the patients with ipsiversive and contraversive tilts of the SVV. Note that each group includes 12 patients with no directional SVV tilt (0°).

^a^Mann–Whitney-U-test.

^b^ *X* ^2^ test.

Distribution of gender showed no significant difference between the groups of ipsiversive and contraversive tilts of SVV (*X*^2^-test, *P* = 0.921). Co-occurrence of contralateral paresis or contralateral somatosensory deficits was high in both groups, whereas spatial neglect (determined as Center of Cancellation > 0.081 in the Bells Test or personal/peri-personal neglect evident on clinical examination), and contraversive pushing occurred in less than a third of patients. The frequency of spatial neglect, contralateral somatosensory deficits or paresis and contraversive pushing was not significantly different between the two groups (Table 1).

### SVR-LSM

**Figure 2:**
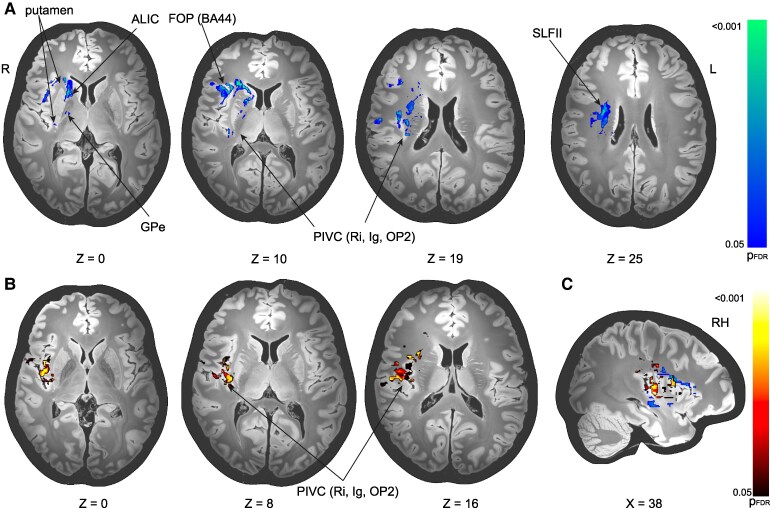
**(A) SVR-LSM of lesions associated with contraversive SVV tilts (in °, *n* = 56). (B) SVR-LSM of lesions associated with ipsiversive SVV tilts (in °, *n* = 60).** A Contraversive tilts shows clusters in the frontal opercular cortex (FOP, Brodman area 44 (BA44), the BG (anterior and posterior putamen, globus pallidus (external part (GPe), and caudate nucleus). Additional clusters were located in the ALIC and SLFII. Furthermore, clusters were found around the PIVC including Ri, Ig, OP2. B SVR-LSM of ipsiversive tilts shows clusters located mainly around the PIVC (areas Ri, Ig, OP2-3). C shows integrated results from A to B. Note that there are distinct but also overlapping clusters associated with ipsiversive (hot colour scheme) and contraversive tilts (cold colour scheme). All results presented with their significance thresholds (*P* < 0.05, FDR-corrected for multiple comparisons after extensive permutation testing (10 000 permutations). SVR-LSM support-vector regression lesion-symptom mapping, Gpe external segment of the globus pallidus, FOP frontal opercular cortex, BA44 Brodman area 44, SLFII 2nd branch of the superior longitudinal fascicle, PIVC parieto-(retro-) insular vestibular cortex, Ri retroinsular cortex, Ig insular posterior long gyrus, OP2 parietal opercular cortex, FDR, false discovery rate; RH, right hemisphere, (FDR-corrected *P*-value).

We found overlapping but also distinct clusters associated with ipsiversive and contraversive tilts of the SVV. Clusters associated with both ipsiversive and contraversive tilts were located in the posterior insula (posterior insular long gyrus) and adjacent opercular cortex (OP2-3, Fig. 2A and B).

The clusters that were associated only with contraversive tilts were located more anteriorly in the frontal operculum centered around the ventrolateral prefrontal cortex (BA44) and subcortically in the globus pallidus, putamen and the anterior limb of the internal capsule (ALIC) as well as in the second branch of the superior longitudinal fascicle (SLFII; Fig. 2A).

Ipsiversive tilt clusters centered around the posterior insula and included all parts of the human homologue of the parieto-(retro-) insular vestibular cortex—area PIVC [areas Ri—retroinsula and OP2-3 (parietal opercular cortex)]. The cluster extended anteriorly towards the mid insula and adjacent operculum. Additional clusters were found in the ventral motor cortex (area 3av, Fig. 2B; Supplementary Fig. 1 shows the results if only patients with SVV tilts <2.5° are considered). C shows integrated results from A to B. Note that there are distinct but also overlapping clusters associated with ipsiversive (hot colour scheme) and contraversive tilts (cold colour scheme).

### SVR-LSM-disconnectome mapping

**Figure 3:**
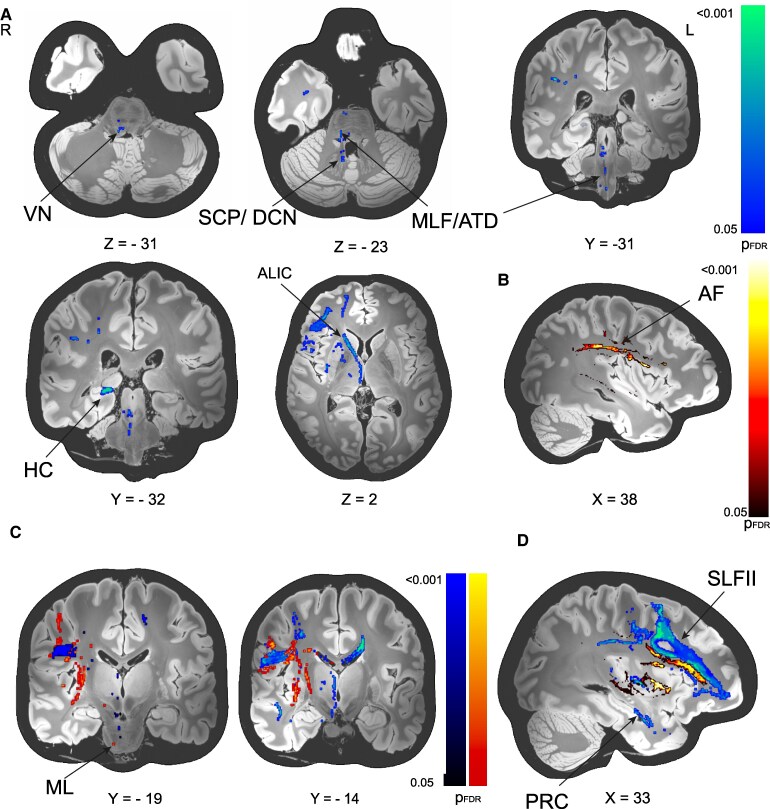
**SVR-LSM using the lesion-based disconnectome maps. (A) The contraversive tilt disconnectome network (SVV tilt in °, *n* = 56). (B) Ipsiversive tilt disconnectome network (SVV tilt in °, *n* = 60). (C) Brainstem disconnection for contraversive and ipsiversive SVV tilts. (D) Separate fronto-parietal disconnection in ipsiversive and contraversive SVV tilts.** A The contraversive tilt disconnectome network showed cortico-fugal disconnection via the thalamus to the VN (via MLF, ATD) and vestibulo-cerebellum via SCP to lobule IX and the DCN. Cortico-cortical disconnection was found for the SLFII and the ALIC with connections to the mesial temporal lobe (PRC and HC). B The ipsiversive tilt disconnectome network included mainly the AF/SLFIII and extreme capsule, providing local connections between insula, operculum, and parietal cortex (IPS). This included connections towards the somatosensory cortical areas as well as towards the intraparietal sulcus. C Coronal depiction of results from A and B for clear visualization of minor clusters in the brainstem (ML, MLF) D Overlay of the results from A and B shows distinct disconnection of fronto-parietal association tracts (SLFII for contraversive tilts, AF/SLFIII for ipsiversive tilts). ALIC anterior limb of the internal capsule, AF arcuate fascicle, ATD ascending Deiters’ tract, ML medial lemniscus, MLF medial longitudinal fascicle, DCN deep cerebellar nuclei, HC hippocampus, SCP superior cerebellar peduncle, SLFIII 3rd branch of the superior longitudinal fascicle, VN vestibular nuclei, PRC peri-rhinal cortex, AF arcuate fascicle, SVR-LSM support-vector regression lesion-symptom mapping, FDR false discovery rate, RH right hemisphere, pFDR (false-discovery rate corrected *P*-value). All results presented with their significance thresholds (*P* < 0.05, FDR-corrected for multiple comparisons after extensive permutation testing (10 000 permutations).

Disconnections of contraversive SVV tilt mediating tracts were located more medially and dorsally around the second branch of the superior longitudinal fascicle (SLFII, Fig. 3A and D). In addition, we detected disconnection of the ALIC and disconnection of the medial temporal lobe, namely the perirhinal/entorhinal cortex. Downstream disconnections could be traced through the thalamus to the midbrain and to the area of the vestibular nuclei (VN) of the pontomedullary brainstem (likely representing the middle longitudinal fascicle/ascending Deiters’ tract and parapontine reticular formation projections) (Fig. 3C). Additional disconnection was observed with the cerebellum via the superior cerebellar peduncle connecting the vestibular cortical network with the deep cerebellar nuclei and lobule IX.

Disconnection of tracts associated with ipsiversive tilt (Fig. 3B) were centered around the PIVC and provide short distance connections via the fronto-insular tracts and longer range temporo-parieto-frontal connections via the arcuate fascicle (AF) and extreme capsule. Brainstem disconnection clusters were sparse and likely located in the medial lemniscus (ML, Fig. 3C).

Contraversive tilt-related disconnection of fronto-parietal association tracts was located more medially and dorsal (SLFII) compared with the disconnection of tracts associated with ipsiversive tilts (AF, Fig. 3D).

### SVR-LSM-functional disconnection

**Figure 4:**
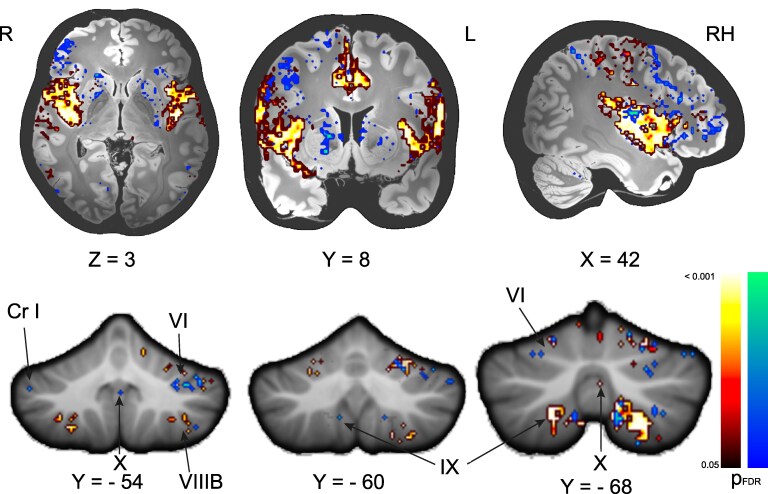
**SVR-LSM using the fc maps for ipsiversive (SVV tilt in °, *n* = 56). Contraversive tilts (SVV tilt in °, *n* = 60).** The ipsiversive tilt network (hot colour scheme) was mainly located around the cortical vestibular network. The contraversive tilt network (cold colour scheme) additionally included the BG and frontal cortex. We found fc with cerebellar motor lobule VI, crus I (Cr I), sensorimotor lobule VIIIB and vestibular lobules IX, X (uvula, nodulus). All results depicted with the significance threshold (*P* < 0.05, FDR-corrected for multiple comparisons after extensive permutation testing (10 000 permutations). SVR-LSM support-vector regression lesion-symptom-mapping, FDR false discovery rate.

The fc maps were used for an SVR-LSM analysis to find associations between SVV tilts and the functional networks. We found an association of the core cortical vestibular network for ipsiversive tilts and additional involvement of the motor system for contraversive tilts (Fig. 4). In addition to these findings, the SVR-LSM analysis for contraversive SVV tilt also revealed cerebello-cortical fc between the motor cerebellum (lobule IV), crus I and sensory and vestibular lobules VIIIB, IX, X (uvula, nodule, flocculus), and the cortex.

## Discussion

The current multimodal lesion-network study differentiated locations and functions of the vestibular cortical network related to gravity perception in the RH. The following findings were established: (i) Separate cortical and subcortical network hubs seem to exist for crossed or uncrossed gravity projections. (ii) Disconnectome mapping provided evidence for the involvement of fronto-temporo-parietal association tracts for uncrossed (AF/SLFIII) and crossed gravity processing (SLFII). (iii) The crossed projections for gravity processing connected the VN and vestibulo-cerebellum with the basal ganglia (BG), the vestibular cortex area PIVC, and the perirhinal cortex. The vestibular connection with the medial temporal lobe could be related to the head direction pathway for spatial navigation that has been established in rodents.^35^ The uncrossed projections could convey vestibular coordinates for head position and sensory postural control. (iv) The functional lesion-network similarly reflected the specific functional network topography of the uncrossed and crossed projections. The functional lesion-networks connect cerebellar network hubs with the cortex.

### Distinct cortical terminations of ipsilateral and contralateral graviceptive pathways

**Figure 5:**
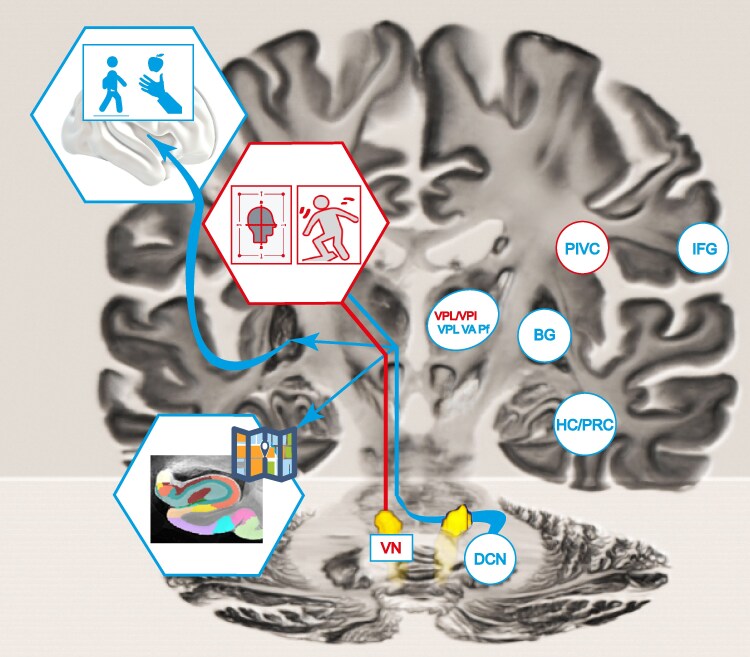
**Functional specialization model of the crossed and uncrossed vestibular pathways.** The bilateral termination in the PIVC could provide a head-centered reference frame for spatial orientation and postural control for a common percept of balance. The crossed projections to the motor system (motor planning) and hippocampus (navigation) could provide graviceptive information for task- and direction-specific movements. The latter seem not to have a bilateral representation. Hippocampal subfield segmentation image from,^40^ histological brain template in MNI space from.^41^ VN vestibular nucleus, DCN deep cerebellar nuclei, BG basal ganglia, VA ventral anterior thalamic nucleus, Pf parafascicular nucleus, VPL/VPI ventral posterior lateral / inferior thalamic nucleus, PIVC parieto-(retro-) insular vestibular cortex, PRC peri-rhinal cortex, HC hippocampus, IFG inferior frontal gyrus.

Vestibular graviception is conveyed via several subcortical pathways to the thalamic nuclei and from there reaches the cortical vestibular network hubs.^7,36,37^ Differential connectivity profiles for ipsilateral and contralateral projections have been established in the brainstem and up to the thalamic level in humans.^6,7,14-16^ The functional connectivity profiles of ipsilateral and contralateral projections via the thalamus suggested a preserved dichotomy of ipsilateral and contralateral gravity projections.^7^ A few studies have used SVV tilts after hemispheric infarcts, to establish the cortical areas that are crucial for verticality perception.^38,39^ While these studies used the absolute tilt of the SVV, the current analysis took the directionality of the tilt into account. With the current data, we could confirm the importance of the PIVC for cortical graviceptive processing as it appeared that PIVC received both crossed and uncrossed projections. In our opinion, the bilateral projections to the PIVC would be integrated for one global percept of the self in space. This could serve as a reference frame for spatial perception. The additional unilateral projections could be used for specific functions. Based on the anatomical location the crossed pathways could be involved in locomotor planning and navigation since they connect with the BG (anterior and posterior putamen, globus pallidus and caudate nucleus) and the ALIC and SLFII. The uncrossed pathway could mediate the multisensory vestibular control of eye, head and body posture by providing local connections between insula, operculum, and parietal cortex, including connections towards the somatosensory cortical areas as well as towards the intraparietal sulcus (Fig. 5).

### Vestibular BG projections

Vestibular projections to the BG are understudied in humans. In rodents, vestibular BG connections to the putamen and caudate nucleus have been demonstrated.^42^ In a prior lesion-mapping effort in humans, vestibular projections to the parafascicular nucleus of the thalamus were detected.^7^ The parafascicular nucleus projects to the BG, which led to the hypothesis that this thalamic network hub is important for vestibular modulation of motor output.^43^ The current data now provide indirect evidence for the importance of the putamen and caudate for the contralateral (crossed) gravity pathway.^7,42,44^ An association of contralateral graviceptive processing with the motor system and frontal cortex (BG, motor and prefrontal cortex) was also observed in the current functional network analysis. The SLFII provides the white matter connection that links parietal projections of the contralateral pathway with the frontal cortex.

### Ascending vestibular projections to thalamo-cortical and limbic networks

Like the BG connections, vestibular projections via the anterior thalamic nuclei to the peri- and enthorinal cortex were shown in rodents.^45-47^ In humans, lesions of the anterior thalamic nuclei are inherently difficult to study because most of these lesions lead to disorders of consciousness. With the current disconnectivity analysis, the gravity network from the VN via the thalamus and BG could be extended to the cortex and the entorhinal/ peri-rhinal cortex (PRC), which could use vestibular signals for heading direction for navigation.^48^ In addition, the functional lesion-network provided evidence for cerebellar connections with the cortical vestibular and motor networks regarding graviceptive processing, namely through connections with motor lobule VI and crus I, sensorimotor lobule VIIIB, and the vestibular lobules IX, X (uvula, nodulus, flocculus).

### Translational use of the gravity network

With the current data, a cortico-subcortical network model for uncrossed graviceptive processing for postural control and crossed graviceptive processing for motor output modulation and navigation could be established in the RH. How might these findings be translated into clinical practice? The perception of gravity relative to the position of the body in the 3-dimensional space is one of the most essential human percepts, as it is crucial for bipedal stance and locomotion.^1^ It seems that the formation of a global reference frame for the self in space relies on both the crossed and uncrossed projections to the cortical vestibular core area, the PIVC. One could probe this concept in a dedicated fMRI study that investigates the proposed networks with specific tasks related to postural control and navigation. Indeed, non-invasive neuromodulation by galvanic vestibular stimulation was tested in patients with progressive supranuclear palsy and shown to improve postural symptoms.^49-51^ Connectivity-based modulation of this network could also improve postural stability in patients with severe postural disorders such as thalamic astasia or contraversive pushing. On the other hand, the more anteriorly crossed graviceptive projection could be especially important for spatial navigation with projections to the PRC.

### Limitations

All disconnectivity analyses were conducted in high-quality tractography and resting state functional connectivity data in healthy adults and therefore only indirectly assess connectomic changes. The data do not demonstrate changes in functional connectivity on the individual patient level. This might present a potential confound because inter-individual variability of the structural and functional connections cannot be studied conclusively. However, the comprehensive study of neurological patient samples is inherently difficult due to the acuity of the disease, limited patient numbers, motion artefacts and the time that patients can lie in the MRI scanner. This is especially true for DWI for tractography, where long scanning times with little head motion are required in addition to the clinical scan. Furthermore, the microenvironment close to the ischaemic lesion presents potential confounders for structural and functional connectivity.^52,53^ The approach using normative connectomes to study network alterations in neurologic patients is widely used and has yielded reliable results in numerous studies.^7,25,54^ Therefore, we are confident that the given limitations are clearly outweighed by the advantages of having high-quality data from large datasets to account for inter-individual differences.

In addition, the voxel-wise statistics on the disconnectome maps have some limitations. We cannot directly assess the affection of specific white matter tracts as multiple crossing or adjacent tracts can pass through a given voxel.

For the SVR-lesion-networks we used the HCP 100 unrelated subjects’ dataset for fcMRI and a separate dataset of 180 subjects with high-quality tractography data at 7T. These data represent curated subsamples of the full HCP dataset and have been used in several studies in the past.^25,55^ The ideal dataset for lesion-network mapping is debated.^56^ Prior studies have shown that using the 100 unrelated subjects’ dataset can provide similar results compared with the full HCP dataset albeit with higher *t*-values in greater sample sizes as results stabilized beyond 150 subjects.^57^ In contrast to prior studies, we additionally performed a SVR with the fc-maps to determine voxel associated with SVV tilts after permutation testing and FDR-correction to account for the lower sample size compared with other studies using the entire HCP dataset.^58,59^

In all SVR analyses, lesion volume was regressed out of behavioural and lesion data. Therefore, these results might be overly conservative. However, alternative analyses regressing lesion volume only in lesion data showed no significant differences in the general pattern of the distribution of significant voxels (see Supplementary Figs 2–4).

So far, we described right-hemisphere lesion-based connectivity networks. Whether this connectivity profile can be translated to left-hemisphere lesions is currently not known. Given the known right-hemispheric dominance for vestibular processing, it would be interesting to see if the same dichotomy can be observed in the left hemisphere as well.

## Conclusions

The current data extend the network for gravity processing from the VN and cerebellum to the cortex of the RH. This network model now includes BG and medial temporal lobe structures, possibly for motor output modulation and navigation. In view of the hemispheric dominance of vestibular networks, a key question for future studies will be whether a similar network organization also exists in the left hemisphere.

## Supplementary Material

fcaf290_Supplementary_Data

## Data Availability

The lesion maps and clinical data are publicly available in the framework of the Open Research Foundation (https://osf.io/udmq3/) upon publication.

## References

[1] Dieterich M, Brandt T. Perception of verticality and vestibular disorders of balance and falls. Front Neurol. 2019;10:172. 31001184 10.3389/fneur.2019.00172 PMC6457206

[2] Cullen KE . The vestibular system: Multimodal integration and encoding of self-motion for motor control. Trends Neurosci. 2012;35(3):185–196. 22245372 10.1016/j.tins.2011.12.001 PMC4000483

[3] Carriot J, Brooks JX, Cullen KE. Multimodal integration of self-motion cues in the vestibular system: Active versus passive translations. J Neurosci. 2013;33(50):19555–19566. 24336720 10.1523/JNEUROSCI.3051-13.2013 PMC3858625

[4] Mackrous I, Carriot J, Jamali M, Cullen KE. Cerebellar prediction of the dynamic sensory consequences of gravity. Curr Biol. 2019;29(16):2698–2710.e4. 31378613 10.1016/j.cub.2019.07.006 PMC6702062

[5] Glasauer S, Dieterich M, Brandt T. Computational neurology of gravity perception involving semicircular canal dysfunction in unilateral vestibular lesions. Prog Brain Res. 2019;248:303–317. 31239142 10.1016/bs.pbr.2019.04.010

[6] Baier B, Conrad J, Stephan T, et al Vestibular thalamus: Two distinct graviceptive pathways. Neurology. 2016;86(2):134–140. 26659130 10.1212/WNL.0000000000002238

[7] Conrad J, Baier B, Eberle L, et al Network architecture of verticality processing in the human thalamus. Ann Neurol. 2023;94(1):133–145. 36966483 10.1002/ana.26652

[8] Elwischger K, Rommer P, Prayer D, Mueller C, Auff E, Wiest G. Thalamic astasia from isolated centromedian thalamic infarction. Neurology. 2012;78(2):146–147. 22205761 10.1212/WNL.0b013e31823efc82

[9] Masdeu JC, Gorelick PB. Thalamic astasia: Inability to stand after unilateral thalamic lesions. Ann Neurol. 1988;23(6):596–603. 2841901 10.1002/ana.410230612

[10] Perennou DA, Mazibrada G, Chauvineau V, et al Lateropulsion, pushing and verticality perception in hemisphere stroke: A causal relationship? Brain. 2008;131(Pt 9):2401–2413. 18678565 10.1093/brain/awn170

[11] Karnath HO, Johannsen L, Broetz D, Kuker W. Posterior thalamic hemorrhage induces “pusher syndrome”. Neurology. 2005;64(6):1014–1019. 15781819 10.1212/01.WNL.0000154527.72841.4A

[12] Rosenzopf H, Klingbeil J, Wawrzyniak M, et al Thalamocortical disconnection involved in pusher syndrome. Brain. 2023;146(9):3648–3661. 36943319 10.1093/brain/awad096

[13] Ticini LF, Klose U, Nagele T, Karnath HO. Perfusion imaging in pusher syndrome to investigate the neural substrates involved in controlling upright body position. PLoS One. 2009;4(5):e5737. 19478939 10.1371/journal.pone.0005737 PMC2684628

[14] Baier B, Thomke F, Wilting J, Heinze C, Geber C, Dieterich M. A pathway in the brainstem for roll-tilt of the subjective visual vertical: Evidence from a lesion-behavior mapping study. J Neurosci. 2012;32(43):14854–14858. 23100408 10.1523/JNEUROSCI.0770-12.2012 PMC6704844

[15] Zwergal A, Buttner-Ennever J, Brandt T, Strupp M. An ipsilateral vestibulothalamic tract adjacent to the medial lemniscus in humans. Brain. 2008;131(Pt 11):2928–2935. 18772222 10.1093/brain/awn201

[16] Yang TH, Oh SY, Kwak K, Lee JM, Shin BS, Jeong SK. Topology of brainstem lesions associated with subjective visual vertical tilt. Neurology. 2014;82(22):1968–1975. 24793187 10.1212/WNL.0000000000000480

[17] Baier B, Cuvenhaus HS, Muller N, Birklein F, Dieterich M. The importance of the insular cortex for vestibular and spatial syndromes. Eur J Neurol. 2021;28(5):1774–1778. 33270346 10.1111/ene.14660

[18] Dichgans J, Held R, Young LR, Brandt T. Moving visual scenes influence the apparent direction of gravity. Science. 1972;178(4066):1217–1219. 4637810 10.1126/science.178.4066.1217

[19] Karnath HO, Ferber S, Dichgans J. The origin of contraversive pushing: Evidence for a second graviceptive system in humans. Neurology. 2000;55(9):1298–1304. 11087771 10.1212/wnl.55.9.1298

[20] Gauthier L, Dehaut F, Joanette Y. The bells test: A quantitative and qualitative test for visual neglect. Int J Clin Neuropsychol. 1989;11(2):49–54.

[21] Rorden C, Karnath HO. A simple measure of neglect severity. Neuropsychologia. 2010;48(9):2758–2763. 20433859 10.1016/j.neuropsychologia.2010.04.018 PMC3129646

[22] DeMarco AT, Turkeltaub PE. A multivariate lesion symptom mapping toolbox and examination of lesion-volume biases and correction methods in lesion-symptom mapping. Hum Brain Mapp. 2018;39(11):4169–4182. 29972618 10.1002/hbm.24289 PMC6647024

[23] Zhang Y, Kimberg DY, Coslett HB, Schwartz MF, Wang Z. Multivariate lesion-symptom mapping using support vector regression. Hum Brain Mapp. 2014;35(12):5861–5876. 25044213 10.1002/hbm.22590 PMC4213345

[24] Edlow BL, Mareyam A, Horn A, et al 7 Tesla MRI of the ex vivo human brain at 100 micron resolution. Sci Data. 2019;6(1):244. 31666530 10.1038/s41597-019-0254-8 PMC6821740

[25] Boes AD, Prasad S, Liu H, et al Network localization of neurological symptoms from focal brain lesions. Brain. 2015;138(Pt 10):3061–3075. 26264514 10.1093/brain/awv228 PMC4671478

[26] Thiebaut de Schotten M, Foulon C, Nachev P. Brain disconnections link structural connectivity with function and behaviour. Nat Commun. 2020;11(1):5094. 33037225 10.1038/s41467-020-18920-9 PMC7547734

[27] Foulon C, Cerliani L, Kinkingnehun S, et al Advanced lesion symptom mapping analyses and implementation as BCBtoolkit. Gigascience. 2018;7(3):1–17. 10.1093/gigascience/giy004 PMC5863218 29432527

[28] Van Essen DC, Smith SM, Barch DM, et al The WU-Minn human connectome project: An overview. Neuroimage. 2013;80:62–79. 23684880 10.1016/j.neuroimage.2013.05.041 PMC3724347

[29] Rojkova K, Volle E, Urbanski M, Humbert F, Dell'Acqua F, Thiebaut de Schotten M. Atlasing the frontal lobe connections and their variability due to age and education: A spherical deconvolution tractography study. Brain Struct Funct. 2016;221(3):1751–1766. 25682261 10.1007/s00429-015-1001-3

[30] Wiesen D, Karnath HO, Sperber C. Disconnection somewhere down the line: Multivariate lesion-symptom mapping of the line bisection error. Cortex. 2020;133:120–132. 33120190 10.1016/j.cortex.2020.09.012

[31] Van Essen DC, Ugurbil K, Auerbach E, et al The human connectome project: A data acquisition perspective. Neuroimage. 2012;62(4):2222–2231. 22366334 10.1016/j.neuroimage.2012.02.018 PMC3606888

[32] Glasser MF, Sotiropoulos SN, Wilson JA, et al The minimal preprocessing pipelines for the Human Connectome Project. Neuroimage. 2013;80:105–124. 23668970 10.1016/j.neuroimage.2013.04.127 PMC3720813

[33] Smith SM, Beckmann CF, Andersson J, et al Resting-state fMRI in the human connectome project. Neuroimage. 2013;80:144–168. 23702415 10.1016/j.neuroimage.2013.05.039 PMC3720828

[34] Whitfield-Gabrieli S, Nieto-Castanon A. Conn: A functional connectivity toolbox for correlated and anticorrelated brain networks. Brain Connect. 2012;2(3):125–141. 22642651 10.1089/brain.2012.0073

[35] Smith PF . The vestibular system and cognition. Curr Opin Neurol. 2017;30(1):84–89. 27845944 10.1097/WCO.0000000000000403

[36] Kirsch V, Keeser D, Hergenroeder T, et al Structural and functional connectivity mapping of the vestibular circuitry from human brainstem to cortex. Brain Struct Funct. 2016;221(3):1291–1308. 25552315 10.1007/s00429-014-0971-x

[37] Lang W, Büttner-Ennever JA, Büttner U. Vestibular projections to the monkey thalamus: An autoradiographic study. Brain Res. 1979;177(1):3–17. 115546 10.1016/0006-8993(79)90914-4

[38] Baier B, Suchan J, Karnath HO, Dieterich M. Neural correlates of disturbed perception of verticality. Neurology. 2012;78(10):728–735. 22357719 10.1212/WNL.0b013e318248e544

[39] Brandt T, Dieterich M, Danek A. Vestibular cortex lesions affect the perception of verticality. Ann Neurol. 1994;35(4):403–412. 8154866 10.1002/ana.410350406

[40] Ravikumar S, Wisse LEM, Lim S, et al Ex vivo MRI atlas of the human medial temporal lobe: Characterizing neurodegeneration due to tau pathology. Acta Neuropathol Commun. 2021;9(1):173. 34689831 10.1186/s40478-021-01275-7 PMC8543911

[41] Amunts K, Lepage C, Borgeat L, et al BigBrain: An ultrahigh-resolution 3D human brain model. Science. 2013;340(6139):1472–1475. 23788795 10.1126/science.1235381

[42] Stiles L, Smith PF. The vestibular-basal ganglia connection: Balancing motor control. Brain Res. 2015;1597:180–188. 25498858 10.1016/j.brainres.2014.11.063

[43] Mandelbaum G, Taranda J, Haynes TM, et al Distinct cortical-thalamic-striatal circuits through the parafascicular nucleus. Neuron. 2019;102(3):636–652 e7. 30905392 10.1016/j.neuron.2019.02.035 PMC7164542

[44] Luo L, Zhu L, Chen L, Zhou Y, Yang R. Case report: Acute vestibular syndrome following a small infarct on the right dorsolateral putamen. Neurol Sci. 2024;45(9):4611–4613. 38691275 10.1007/s10072-024-07552-2

[45] Taube JS . The head direction signal: Origins and sensory-motor integration. Annu Rev Neurosci. 2007;30:181–207. 17341158 10.1146/annurev.neuro.29.051605.112854

[46] Taube JS . Head direction cells recorded in the anterior thalamic nuclei of freely moving rats. J Neurosci. 1995;15(1 Pt 1):70–86. 7823153 10.1523/JNEUROSCI.15-01-00070.1995 PMC6578288

[47] Winter SS, Clark BJ, Taube JS. Spatial navigation. Disruption of the head direction cell network impairs the parahippocampal grid cell signal. Science. 2015;347(6224):870–874. 25700518 10.1126/science.1259591 PMC4476794

[48] Jacob PY, Poucet B, Liberge M, Save E, Sargolini F. Vestibular control of entorhinal cortex activity in spatial navigation. Front Integr Neurosci. 2014;8:38. 24926239 10.3389/fnint.2014.00038 PMC4046575

[49] Wuehr M, Peto D, Fietzek UM, et al Low-intensity vestibular noise stimulation improves postural symptoms in progressive supranuclear palsy. J Neurol. 2024;271(7):4577–4586. 38722328 10.1007/s00415-024-12419-9 PMC11233287

[50] Wuehr M, Schmidmeier F, Katzdobler S, Fietzek UM, Levin J, Zwergal A. Effects of low-intensity vestibular noise stimulation on postural instability in patients with Parkinson's disease. J Parkinsons Dis. 2022;12(5):1611–1618. 35491798 10.3233/JPD-213127

[51] Duncan SJ, Marques K, Fawkes J, Smith LJ, Wilkinson DT. Galvanic vestibular stimulation modulates EEG markers of voluntary movement in Parkinson's disease. Neuroscience. 2024;555:178–183. 39074577 10.1016/j.neuroscience.2024.07.048

[52] Siegel JS, Snyder AZ, Ramsey L, Shulman GL, Corbetta M. The effects of hemodynamic lag on functional connectivity and behavior after stroke. J Cereb Blood Flow Metab. 2016;36(12):2162–2176. 26661223 10.1177/0271678X15614846 PMC5363662

[53] Jellison BJ, Field AS, Medow J, Lazar M, Salamat MS, Alexander AL. Diffusion tensor imaging of cerebral white matter: A pictorial review of physics, fiber tract anatomy, and tumor imaging patterns. AJNR Am J Neuroradiol. 2004;25(3):356–369. 15037456 PMC8158568

[54] Hollunder B, Ostrem JL, Sahin IA, et al Mapping dysfunctional circuits in the frontal Cortex using deep brain stimulation. Nat Neurosci. 2024;27(3):573–586. 38388734 10.1038/s41593-024-01570-1 PMC10917675

[55] Fischer DB, Boes AD, Demertzi A, et al A human brain network derived from coma-causing brainstem lesions. Neurology. 2016;87(23):2427–2434. 27815400 10.1212/WNL.0000000000003404 PMC5177681

[56] Sperber C, Dadashi A. The influence of sample size and arbitrary statistical thresholds in lesion-network mapping. Brain. 2020;143(5):e40. 32365360 10.1093/brain/awaa094

[57] Cohen AL, Fox MD. Reply: The influence of sample size and arbitrary statistical thresholds in lesion-network mapping. Brain. 2020;143(5):e41. 32365379 10.1093/brain/awaa095 PMC7241951

[58] Joutsa J, Horn A, Hsu J, Fox MD. Localizing parkinsonism based on focal brain lesions. Brain. 2018;141(8):2445–2456. 29982424 10.1093/brain/awy161 PMC6061866

[59] Joutsa J, Shih LC, Fox MD. Mapping holmes tremor circuit using the human brain connectome. Ann Neurol. 2019;86(6):812–820. 31614012 10.1002/ana.25618 PMC6899700

