# Supplementary material for: Structural and functional connectivity of vestibular graviceptive to sensory and motor circuits
Source: Brain Commun. 2025 Aug 8;7(4):fcaf290. doi: 10.1093/braincomms/fcaf290 (PMC12378872; doi:10.1093/braincomms/fcaf290)
Supplement: fcaf290_Supplementary_Data [file fcaf290_supplementary_data.docx]

Supplementary Material: Conrad et al. *Structural and functional connectivity of vestibular graviceptive to sensory and motor circuits.*

*
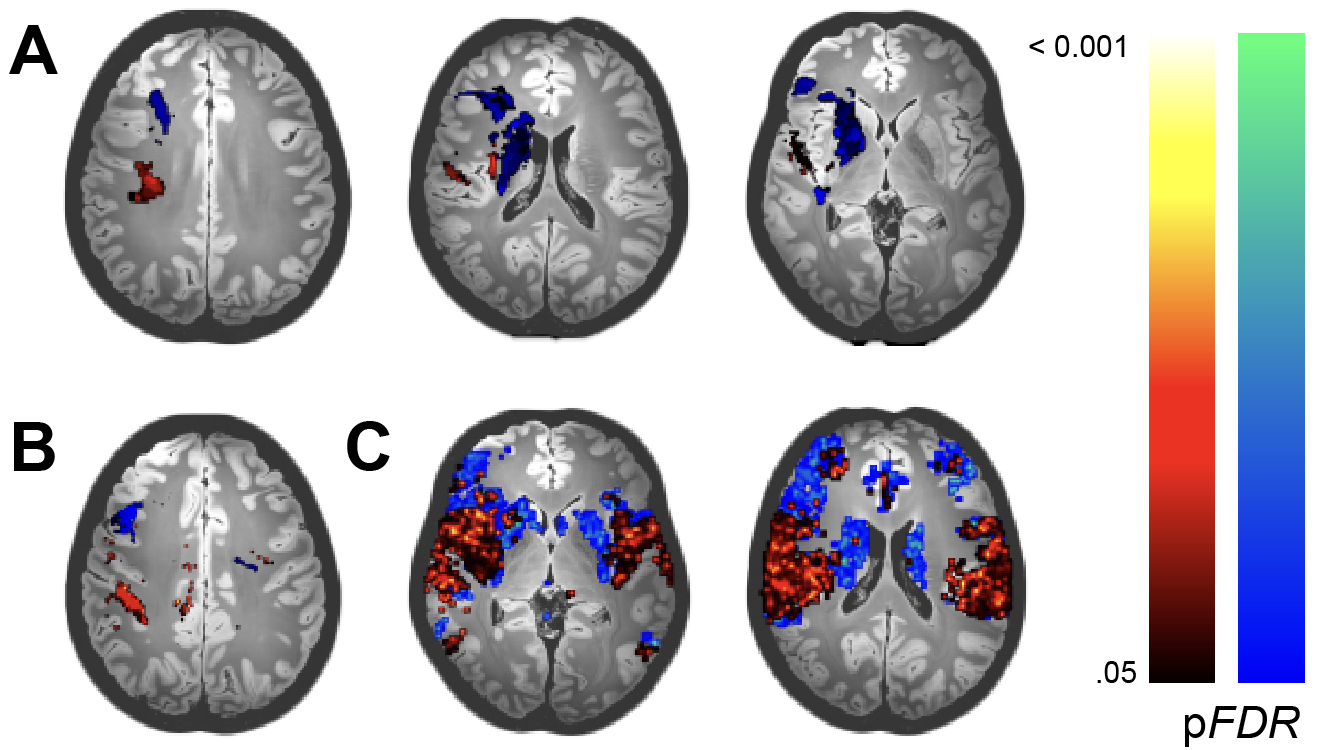
*

**Supplementary Figure 1** SVR-LSM with patients with SVV values above the threshold of +/-2.5° A: SVRLSM Analysis of all cases with pathological SVV tilt (i.e., >2.5°; n=43).

Blue contraversive tilts (n=22), red ipsiversive tilts (n=21).

**B** Disconnectome map SVRLSM shows distinct clusters associated with contraversive (blue) and ipsiversive tilts (red). **C** Fc-SVRLSM also shows diverging fc-patterns for contraversive (blue; basal ganglia, frontal) and ipsiversive networks (hot; insular/opercular).

All SVRLSM-results FDR-corrected using the FDR-function in FSL after extensive permutation testing (10,000 permutations).

Supplementary Table 1: p-value cut-offs for FDR-correction in FSL (q .05, i.e., p<0.05 FDR-corrected).

| **Method** | **Analysis** | **p-value cut-off** |
| --- | --- | --- |
| SVR-LSM | iSVV-SVRLSM | 0.049495 |
|  | cSVV_SVRLSM | 0.049495 |
| Disconnectome-SVRLSM: | cSVV_BCBSVRLSM | 0.0483952 |
|  | iSVV_BCBSVRLSM | 0.0482952 |
| fc_SVRLSM | cSVV_fcSVRLSM | 0.0436956 |
|  | iSVV_fcSVRLSM | 0.0445955 |

**Supplementary Figure 2 Effects of lesion volume control on the SVRLSM (support-vector regression lesion symptom mapping) results (n=56 for ipsiversive tilts, n=60 for contraversive tilts) A & B** using DTLVC (direct total lesion volume control)^1,2^, **C & D** using lesion regression as implemented in the SVR-LSM-toolbox^1^.

**A & C** Contraversive tilts **B & D** ipsiversive tilts. Blue color scheme contraversive tilts, hot color scheme ipsiversive tilts. All SVRLSM-results FDR-corrected using the FDR-function in FSL after extensive permutation testing (10,000 permutations).


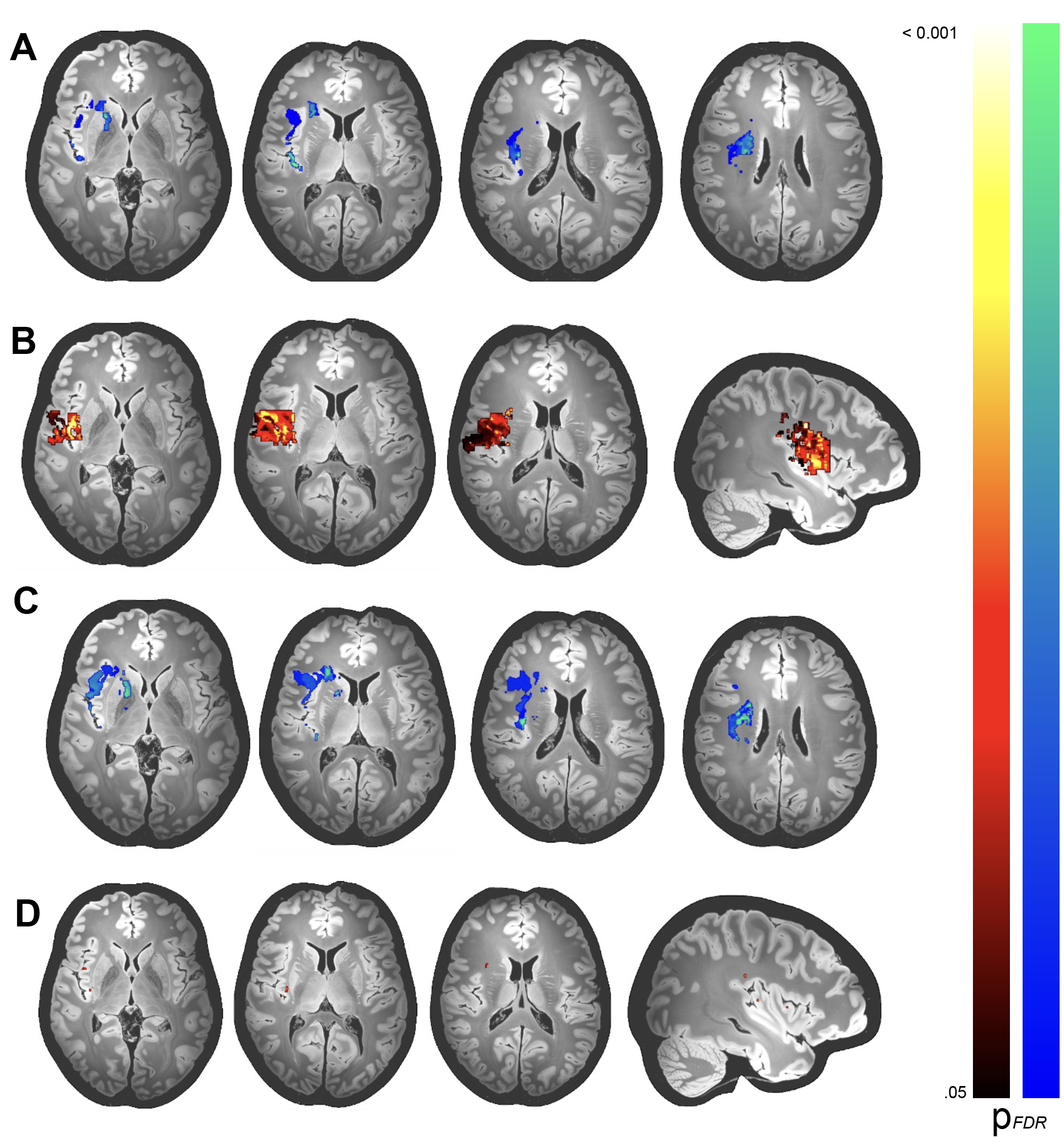


**Supplementary Figure 3 Effects of lesion volume control on the disconnectome SVRLSM (support-vector regression lesion symptom mapping) results (n=56 for ipsiversive tilts, n=60 for contraversive tilts) A, B, C** using DTLVC (direct total lesion volume control)^1,2^, **D, E** using lesion regression as implemented in the SVR-LSM-toolbox^1^. Blue color scheme contraversive tilts, hot color scheme ipsiversive tilts. All SVRLSM-results FDR-corrected using the FDR-function in FSL after extensive permutation testing (10,000 permutations).


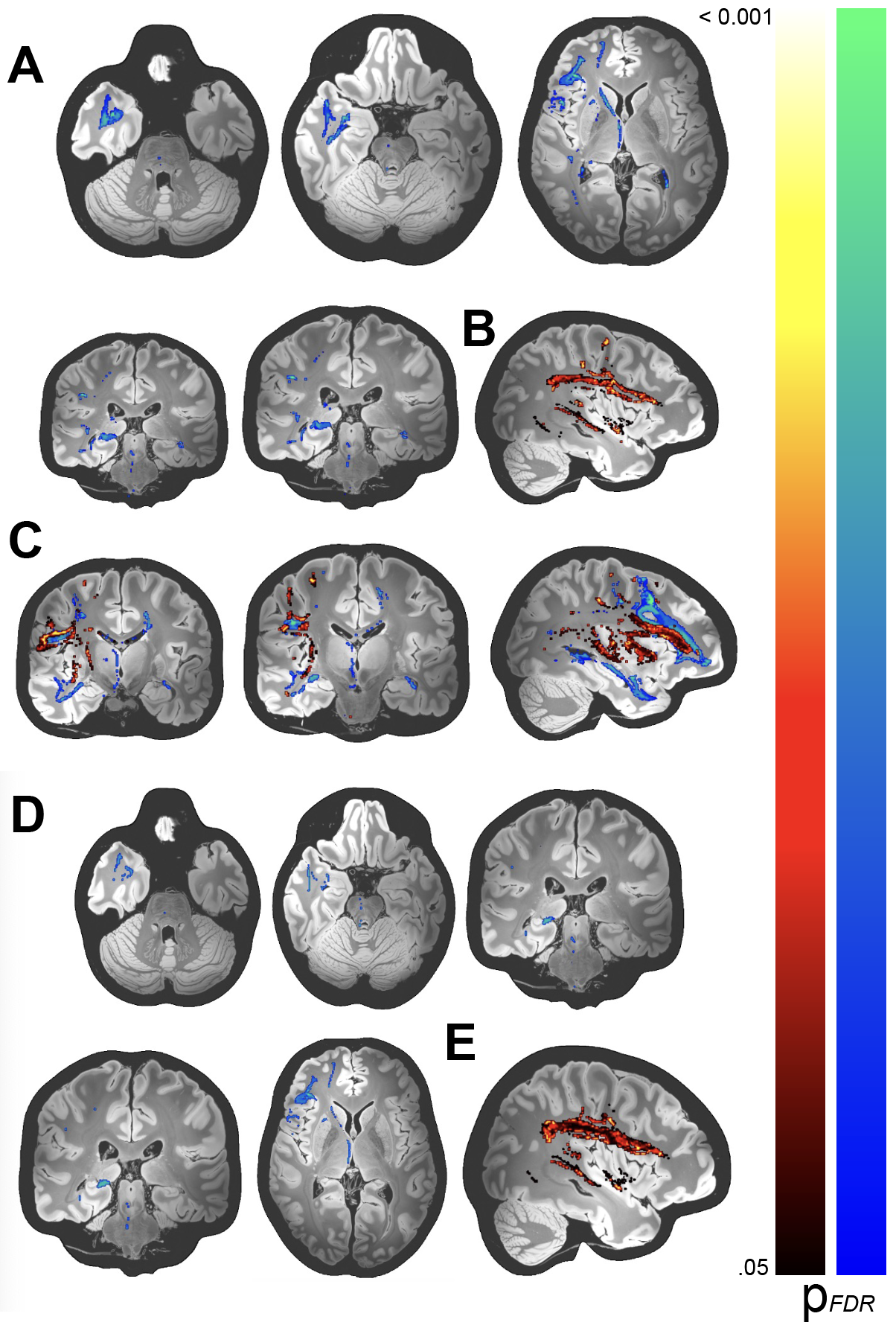


**Supplementary figure 4 Effects of lesion volume control on the functional connectivity suppor-vector regression lesion symptom mapping (fcMRI-SVRLSM) results** (n=56 for ipsiversive tilts, n=60 for contraversive tilts) **A** using DTLVC (direct total lesion volume control)^1,2^, **B** using lesion regression as implemented in the SVR-LSM-toolbox^1^. Blue color scheme contraversive tilts, hot color scheme ipsiversive tilts. All SVRLSM-results FDR-corrected using the FDR-function in FSL after extensive permutation testing (10,000 permutations).


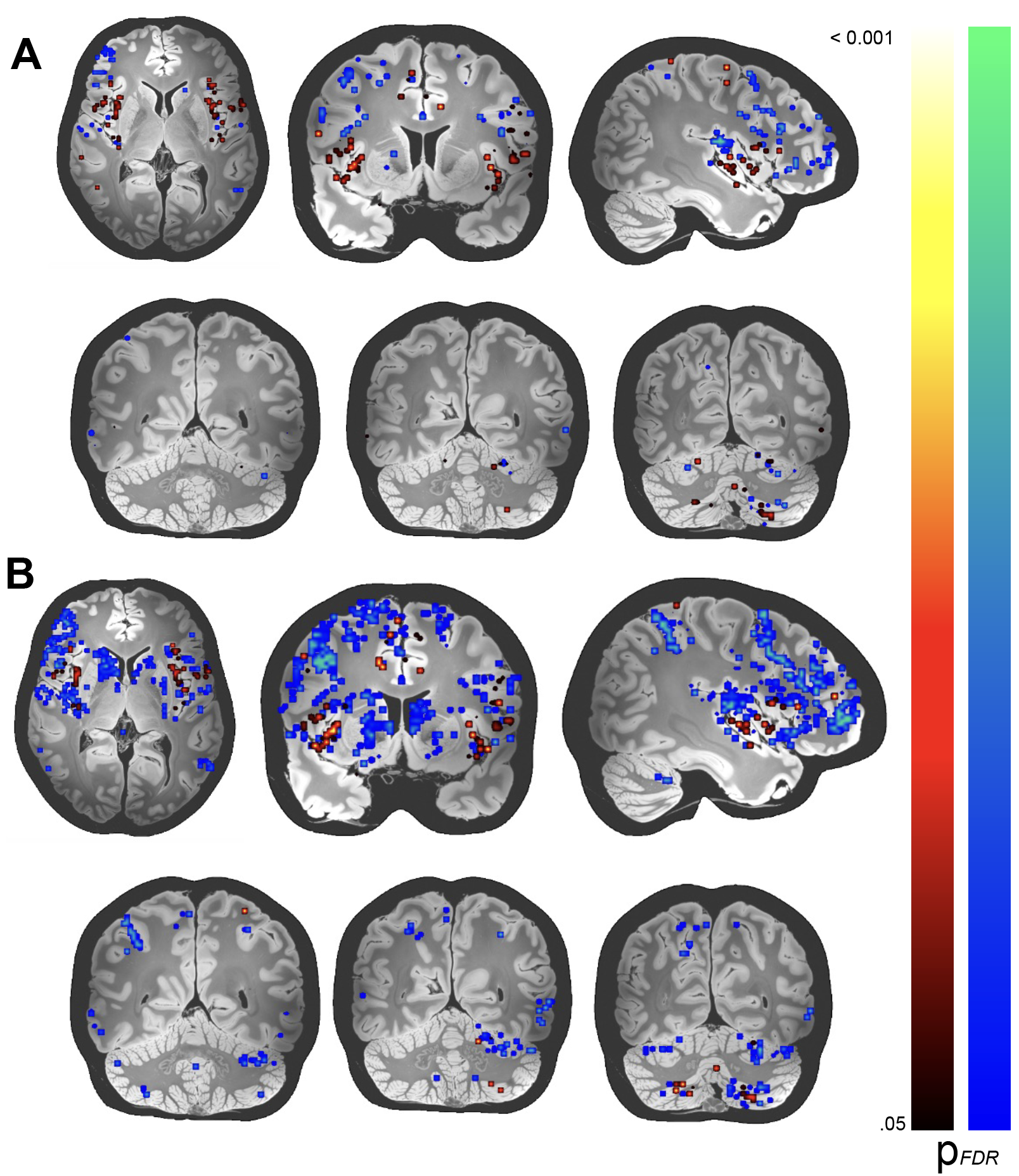


**Supplementary figure 5** Overlap of normative tractography in DSI-studio (<https://dsi-studio.labsolver.org/>) for the proposed white matter tracts with the disconnectome-SVR-LSM results. The tractography results are based on a population based tractography atlas from the human connectome project (n=1065)^3,4^.

**A** Overlap of the significant clusters from the disconnectome SVR-LSM using contraversive tilts with normative tractography using DSI studio. Note the high overlap of these clusters with the SLFII.

**B & C** Overlap of the significant clusters from the SVR-LSM using ipsiversive tilts with normative tractography using DSI studio. Note the high overlap of these clusters with the SLFIII (B) and arcuate fascicle (C). Binarized FDR-corrected SVRLSM results after extensive permutation testing (10,000 permutations).


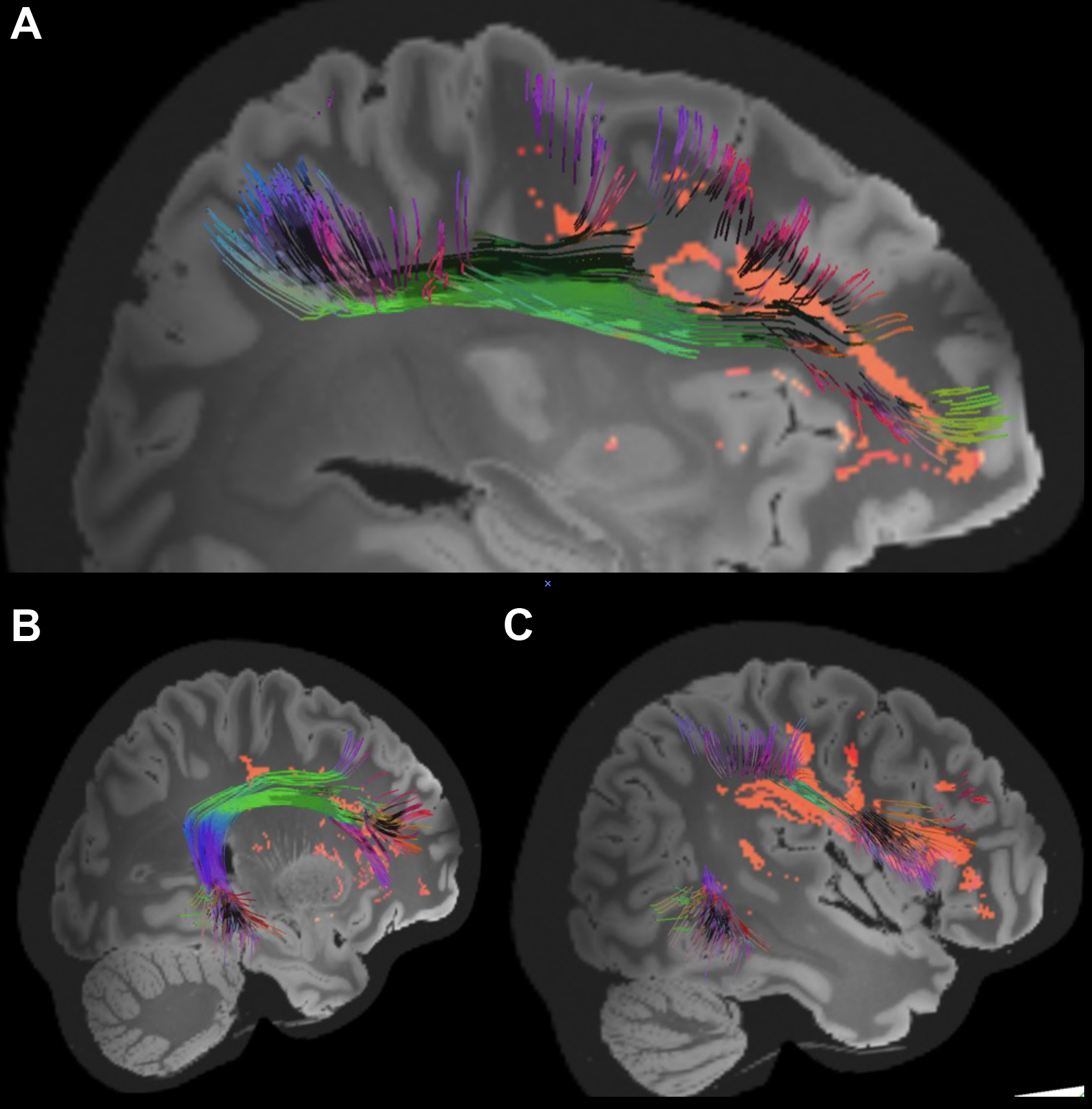


References:

1 DeMarco, A. T. & Turkeltaub, P. E. A multivariate lesion symptom mapping toolbox and examination of lesion-volume biases and correction methods in lesion-symptom mapping. *Hum Brain Mapp* **39**, 4169-4182 (2018). <https://doi.org/10.1002/hbm.24289>

2 Zhang, Y., Kimberg, D. Y., Coslett, H. B., Schwartz, M. F. & Wang, Z. Multivariate lesion-symptom mapping using support vector regression. *Hum Brain Mapp* **35**, 5861-5876 (2014). <https://doi.org/10.1002/hbm.22590>

3 Yeh, F. C. Population-based tract-to-region connectome of the human brain and its hierarchical topology. *Nat Commun* **13**, 4933 (2022). <https://doi.org/10.1038/s41467-022-32595-4>

4 Yeh, F. C. *et al.* Population-averaged atlas of the macroscale human structural connectome and its network topology. *Neuroimage* **178**, 57-68 (2018). <https://doi.org/10.1016/j.neuroimage.2018.05.027>
